# Plant Functional Traits Are Associated with Canopy-to-Soil Hydrological Regulation in Tropical Forests: Evidence from Smallholder Plantations and Secondary Forests

**DOI:** 10.3390/plants15213307

**Published:** 2026-10-26

**Authors:** Zhihui Zhao, Renjie Guo, Zifan Zhang, Wenyang Cao, Lijun Liu, Bingbing Duan, Pei Wang

**Affiliations:** 1 The State Key Laboratory of Earth Surface Processes and Hazards Risk Governance, Faculty of Geographical Science, Beijing Normal University, Beijing 100875, China; 202431051062@mail.bnu.edu.cn (Z.Z.); rjguo1998@163.com (R.G.); zfzhang97@mail.bnu.edu.cn (Z.Z.); 202631051078@mail.bnu.edu.cn (W.C.); 202421051137@mail.bnu.edu.cn (L.L.); 202421051135@mail.bnu.edu.cn (B.D.); 2 School of Natural Resources, Faculty of Geographical Science, Beijing Normal University, Beijing 100875, China

**Keywords:** plant functional traits, canopy structural complexity, plant–soil feedback, forest restoration, forest ecohydrology

## Abstract

**Highlights:**

- Bark roughness, fine-root allocation and root–water matching form a coupled management–trait syndrome.
- Plantations channel ≈3× more rainfall to stemflow (21% vs. 7%) yet infiltrate ≈16× more slowly.
- Stemflow per unit DBH rises ≈6.3× faster in plantations (4.49 vs. 0.71 mm tree^−1^ cm^−1^).
- Root–water matching (0.89 vs. 0.41) is proposed as a candidate restoration indicator (MC ≥ 0.80).

**Abstract:**

Tropical forest conversion to smallholder plantations alters plant community structure, but the coupled eco-hydrological consequences of trait simplification remain poorly characterised. We compared paired smallholder plantation and secondary forests in Hainan, China (*n* = 3 plots per type, one-year campaign) by jointly measuring rainfall partitioning, soil physico-chemical properties, steady-state infiltration, fine-root biomass, understory diversity (H′), bark roughness, DBH, LAI and canopy density. Two robust descriptive patterns emerged: smallholder plantations showed approximately 3-fold higher stand-scale stemflow fraction (≈21% vs. 7%) and markedly lower steady-state infiltration than secondary forests, while the DBH–stemflow slope was about 6-fold steeper in the smallholder plantation. Secondary forests concentrated more fine-root biomass in the upper 0–20 cm layer (68% vs. 42% of total) and exhibited higher root–water matching (0.89 vs. 0.41). The four variables implicated in the proposed aboveground route co-varied along the expected sequence (r = 0.78, 0.82, 0.69 and 0.74), but no association reached significance; with only six plot-level observations we report them descriptively rather than as a fitted path model. We propose a coupled management–trait syndrome framework to organise trait-based restoration thinking beyond canopy cover, and frame candidate—illustrative, not statistically derived—indicators (understory H′ ≥ 1.5; BRI ≥ 2.0; MC ≥ 0.80) as candidate indicators for future validation in factorial experiments that cross weeding frequency with species mixtures. Given the paired two-site design (*n* = 3 plots per type), a ~500 mm site-rainfall difference, together with four non-significant co-variation coefficients, the present contribution is explicitly a hypothesis-generating paired baseline rather than a confirmatory test—providing (i) a multi-indicator reference dataset, (ii) a reusable root–water matching metric, and (iii) candidate restoration indicators for future validation within a tropical Plant–Water–Nutrient programme.

## 1. Introduction

Tropical forests regulate global carbon and hydrological cycling [1], yet extensive areas of species-rich primary forest have been converted to rubber (*Hevea brasiliensis*) and betel nut (*Areca catechu*) mixed smallholder plantations [2,3,4]. Such conversion simplifies community composition and key functional traits, degrading ecosystem functions [5]. How trait simplification cascades through canopy-to-soil hydrological regulation remains poorly understood, which limits ecohydrological restoration of degraded tropical landscapes.

Plant functional traits (PFTs) provide a mechanistic link between community composition and ecosystem processes [6]. Under the Leaf Economics Spectrum (LES) [7] and Root Economics Space (RES) [8], acquisitive syndromes (rapid growth and large diameter at breast height) are traded off against conservative syndromes (dense tissues and structural investment). Plantations concentrate acquisitive syndromes, whereas species-rich secondary forests spread trait syndromes across the LES–RES plane, favoring functional complementarity [5]. We use the understory Shannon–Wiener index (H′; 4.2) as a proxy for trait portfolio richness; “plant diversity” hereafter refers to understory H′, not overstory tree richness.

Aboveground, bark and canopy morphology are associated with rainfall partitioning [9,10]. Canopy density and LAI modulate interception and throughfall energy: multi-tiered canopies dissipate raindrop kinetic energy and yield more uniform drip points [11], while single-layer plantations deliver high-energy throughfall that promotes surface crusting. Smooth, low-Bark-Roughness-Index (BRI) bark lowers the stemflow threshold and funnels water into localized stem-base hotspots [12,13], whereas fissured bark of late-successional species imparts resistance and promotes diffuse wetting [14]; bark water-holding capacity (BWHC, not measured here) is discussed qualitatively only.

Belowground, fine-root biomass (FRB, measured here) is associated with soil structure through biopore formation and aggregate stabilization [15,16]. Surface-concentrated FRB and diverse litter inputs in secondary forests enhance soil organic matter, lower bulk density and elevate steady-state infiltration [17,18]; root length density (RLD) and specific root length (SRL) are treated as literature-based contextual proxies rather than measured variables. In the plantations, reduced litter and FRB redistribution decouple infiltrated water from the active rhizosphere, storing it in deeper micropores (30–60 cm), a root–water mismatch.

Most prior studies in this ecosystem have examined canopy processes and soil properties separately rather than integrating them through a trait-based intermediate-state framework. In this study we contribute three things: (i) a paired-comparison dataset in tropical Hainan that jointly measures bark roughness, rainfall partitioning, soil hydrology, fine-root biomass and understory diversity in the same plots; (ii) a proposed synthetic metric, the root–water matching coefficient, that quantifies the vertical congruence between fine-root distribution and plant-available water across the soil profile; and (iii) a coupled management–trait syndrome framing that organises the joint effects of intrinsic plant traits and silvicultural management on ecohydrological function. We acknowledge from the outset that intrinsic trait effects and management effects are intrinsically confounded in any single-site comparison of plantation and standage, and we treat this confounding as a feature of the framework rather than a flaw to be statistically removed. The present manuscript therefore offers a hypothesis-generating co-variation characterisation of the coupled pattern, intended as a framework and pre-registered map for subsequent larger-scale tests, not as a confirmatory test of causal pathways.

## 2. Results

### 2.1. Rainfall Partitioning Differences

**Figure 1:**
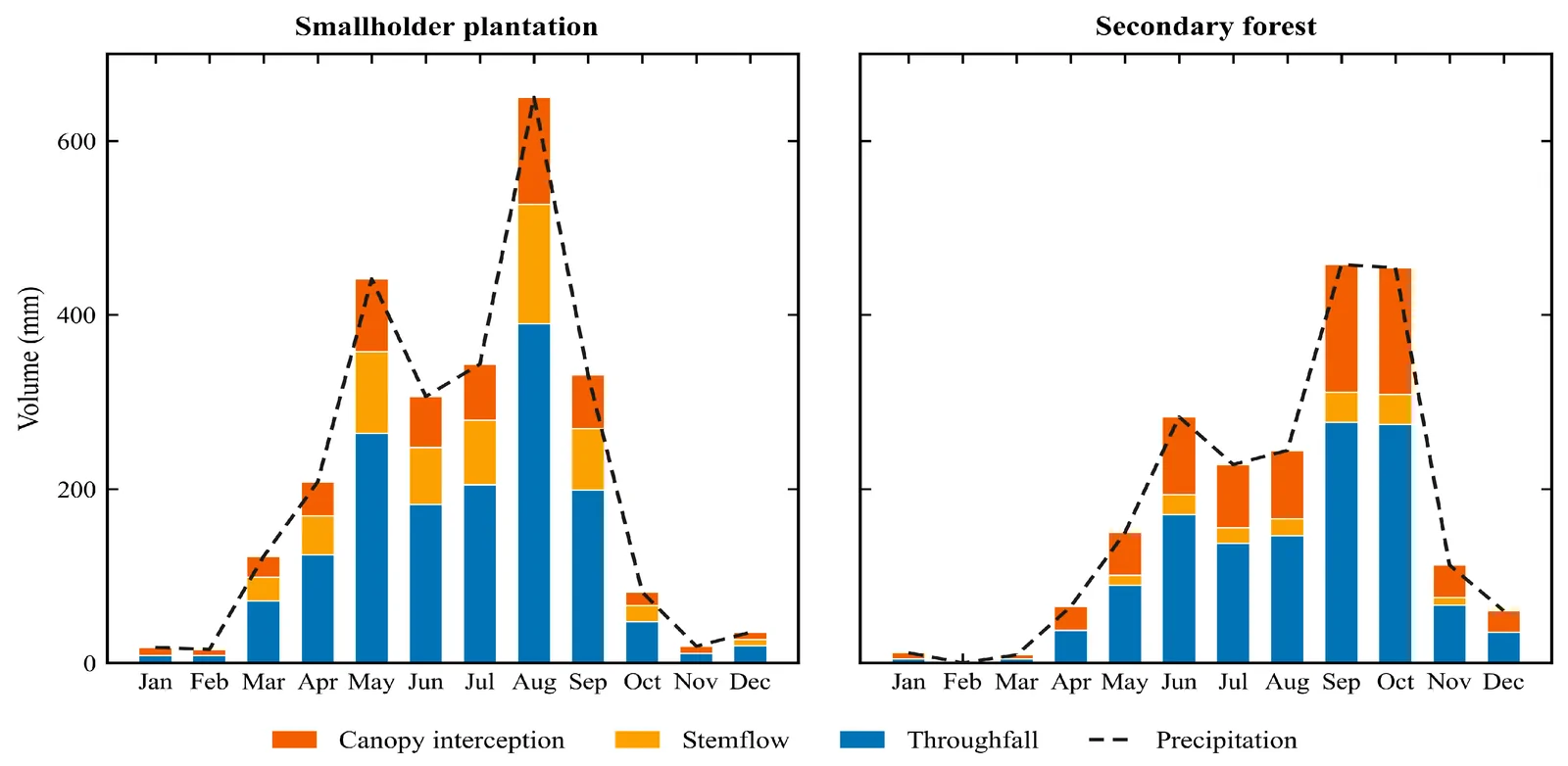
Monthly variation in rainfall partitioning in plantation and secondary forests. Values are means ± SE (error bars denote ± SE); given *n* = 3 plots per type, no significance thresholds are applied.

Rainfall partitioning, encompassing canopy interception (CI), throughfall (TF) and stemflow (SF), differed between the two forest types, with large descriptive contrasts in CI and SF proportions, whereas TF proportions were similar in magnitude (Figure 1; Table 1). Secondary forests intercepted 33.00% of annual rainfall, compared with 19.61% in plantations, consistent with their multi-layered canopy (density 60 ± 3%) and heterogeneous crown architecture dominated by native species such as *Hopea hainanensis* and *Sindora glabra*. Plantations, composed of *Hevea brasiliensis* and *Areca catechu* with a simplified canopy (density 40 ± 2%), lacked this structural complexity and retained less rainfall.

Conversely, monthly SF dynamics displayed the opposite trend (Figure 1): plantations generated a substantially higher SF proportion (20.96 ± 2.45% annually; Table 1) than secondary forests (7.18 ± 2.95%). A slope-based comparison of stemflow generation efficiency per unit DBH revealed a fundamental hydraulic divergence (ANCOVA in 2.2), showing that for a given trunk size rubber trees channel more rainwater into stemflow than do native species. This reflects morphological differences—smooth bark and steep branch angles in *Hevea brasiliensis* versus rough, fissured bark and irregular branching in *Hopea hainanensis*—rather than an artifact of rainfall input magnitude.

TF accounted for 59.45 ± 2.15% (1528.5 ± 28.01 mm) of total rainfall in plantations and 59.81 ± 4.46% (1241.2 ± 34.78 mm) in secondary forests (Table 1), and the difference between the two forest types was negligible (t = 0.32, *p* = 0.76; reported for completeness only—with *n* = 3 plots per type this test has ≈16% power to detect Cohen’s d = 1, so a null result carries little evidential weight; see Section 4.4), consistent with the limited structural contrast in throughfall-funneling canopy geometry between the two forest types.

### 2.2. Trait–Function Relationships: DBH and Bark Roughness Are Associated with Rainfall Partitioning

To quantify the direct association of plant morphological traits with rainfall partitioning, we analyzed the relationships between diameter at breast height (DBH) and stemflow per tree, as well as between bark roughness index (BRI) and throughfall uniformity (TU).

**Figure 2:**
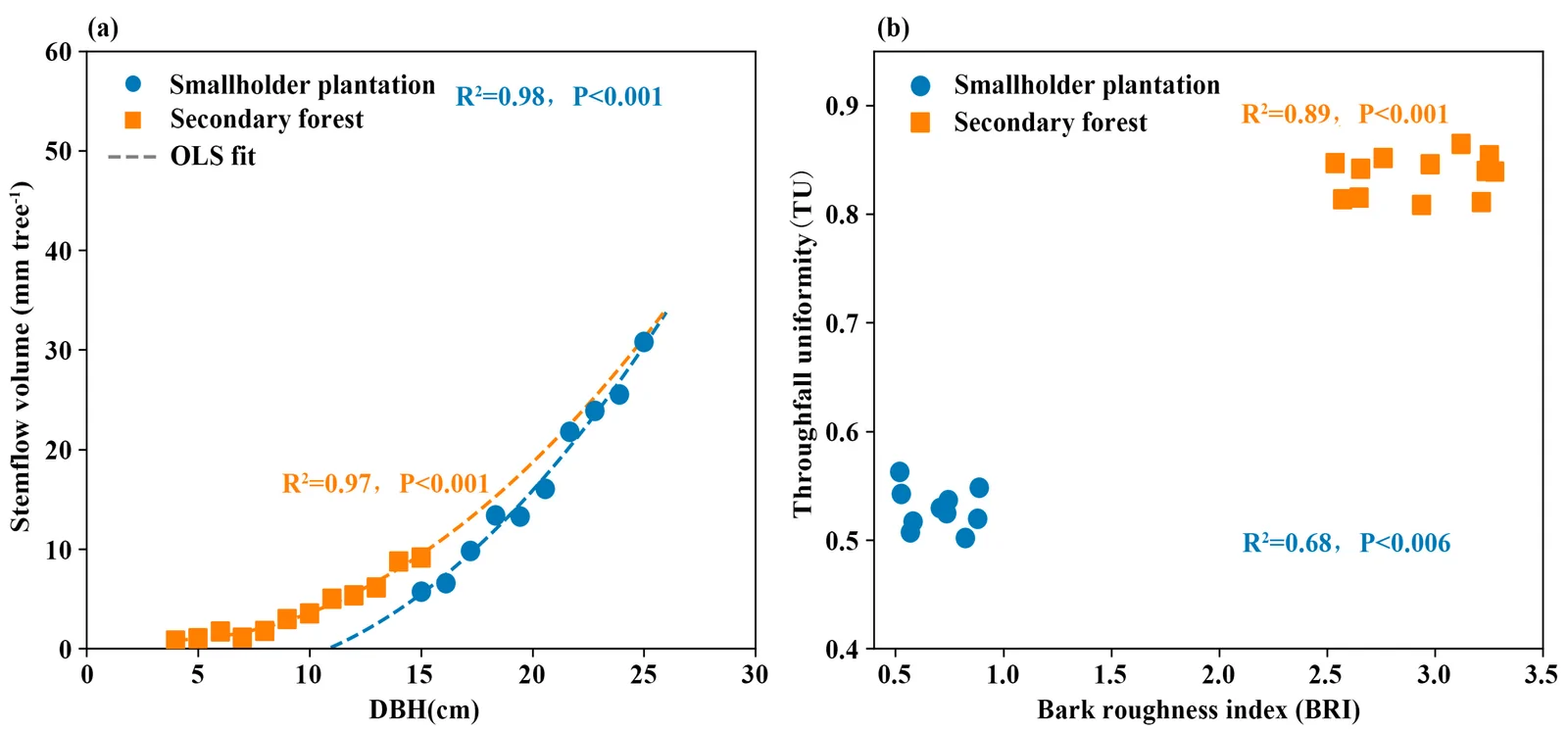
Trait−function relationships between canopy structural/bark traits and localized hydrological partitioning across plantation and secondary forests: (**a**) association of DBH with stemflow volume (linear regression for the plantation, ANCOVA−adjusted slope for the secondary forest); (**b**) influence of bark roughness index (BRI) on throughfall uniformity (TU). Dashed lines denote linear/ANCOVA regressions for plantation (blue) and secondary (orange) forests.

DBH vs. Stemflow per Tree: As illustrated in Figure 2a, stemflow per tree increased consistently and steeply with DBH for both forest types (plantation: R^2^ = 0.98; secondary forest: R^2^ = 0.97). However, the slope of this relationship differed markedly between forest types (analysis of covariance, ANCOVA). Because individual trees, not plots, are the units of these regressions (*n* = 10 trees per forest type), these tree-level regressions are descriptive of the trait–function relationship and are not carried forward as plot-level inference (4.4). In the plantation, the fitted slope was 4.49 mm·tree^−1^·cm^−1^, more than six times that of the secondary forest (0.71 mm·tree^−1^·cm^−1^). The slope expresses the equivalent stemflow depth (mm) generated per tree per cm increase in DBH. At a given DBH, rubber trees generated 2.1–3.5 times more stemflow than secondary forest trees. This difference reflects the smooth bark and steep branch angles of *Hevea brasiliensis*, which efficiently channel rainfall toward the trunk, whereas the rough bark of native species such as *Hopea hainanensis* increases flow resistance and reduces stemflow generation.

Bark Roughness vs. Throughfall Uniformity: The BRI of secondary forest dominants (2.8 ± 0.3) was four times higher than that of rubber trees (0.7 ± 0.1). Correspondingly, throughfall uniformity (TU) was higher in secondary forests (0.81 ± 0.04) than in plantations (0.52 ± 0.05). As shown in Figure 2b, BRI was positively correlated with TU across both forest types (plantation: R^2^ = 0.68; secondary forest: R^2^ = 0.89); these are tree-level correlations (*n* = 10 trees per forest type) and are likewise reported descriptively rather than as plot-level inference. Both plot-level contrasts are reported as effect sizes with 95% confidence intervals instead of significance thresholds, given *n* = 3 plots per forest type (see Section 4.4). In plantations, low BRI allowed rainfall to concentrate rapidly into stemflow, creating pronounced “wet spots” around tree trunks (CV = 34.2%), whereas high BRI in secondary forests promoted spatially uniform throughfall distribution (CV = 18.5%).

### 2.3. Soil Physicochemical Properties and Hydrologic Functions

Soil physicochemical properties varied along the 0–60 cm profile, but contrasts between the two forest types were largely confined to the surface layer (Figure S3). SOM decreased with depth in both forests (from ~24–30 g kg^−1^ at 0–10 cm to ~11–14 g kg^−1^ at 50–60 cm) but showed no detectable forest-type × depth signal (mixed-model Wald F-tests, P > 0.05; Section 4.4); understory H′, available P and N, total N and C likewise did not diverge along the profile (*p* > 0.05). The strongest contrasts were confined to the 0–10 cm layer: the secondary forest had lower bulk density (1.03 vs. 1.24 g cm^−3^) and correspondingly higher total porosity (57.9% vs. 49.4%) than the plantation (effect sizes reported in place of significance thresholds given *n* = 3 plots per type; Figure S3). Below 10 cm, the two forest types shared letters at every depth for bulk density, porosity and SOM.

**Figure 3:**
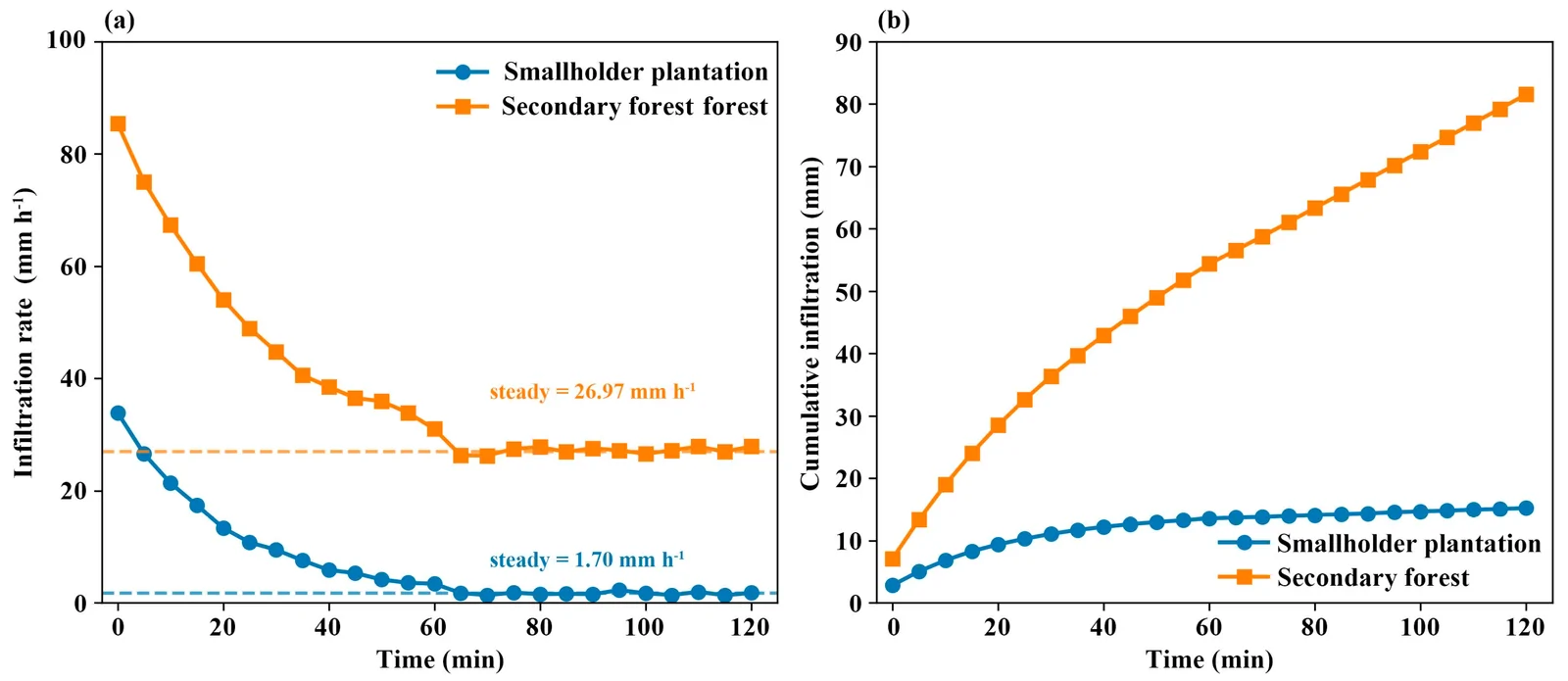
Infiltration rate (**a**) and cumulative infiltration (**b**) over 120 min in plantation and secondary forests. Dashed lines in (**a**) indicate steady−state infiltration rates (plantation ≈ 1.7 mm·h^−1^; secondary ≈ 27.0 mm h^−1^). Values are means ± SE (*n* = 3 plots per type).

Infiltration capacity differed markedly between the two forest types (Figure 3). Over the 120-min measurement, cumulative infiltration in secondary forests reached ≈ 76 mm, about five times that in plantations (≈15 mm; Figure 3b). The steady-state infiltration rate was 26.97 mm·h^−1^ in secondary forests versus 1.70 mm·h^−1^ in plantations—a ≈ 16-fold difference (Figure 3a). In plantations, infiltration declined steeply from ≈ 32 mm·h^−1^ within the first ≈ 30 min to < 10 mm·h^−1^ and approached steady state after ≈ 50–60 min; secondary forests started at ≈ 85 mm·h^−1^, declined more gradually and stabilized at 26.97 mm·h^−1^ after ≈ 60–70 min (Figure 3a).

**Figure 4:**
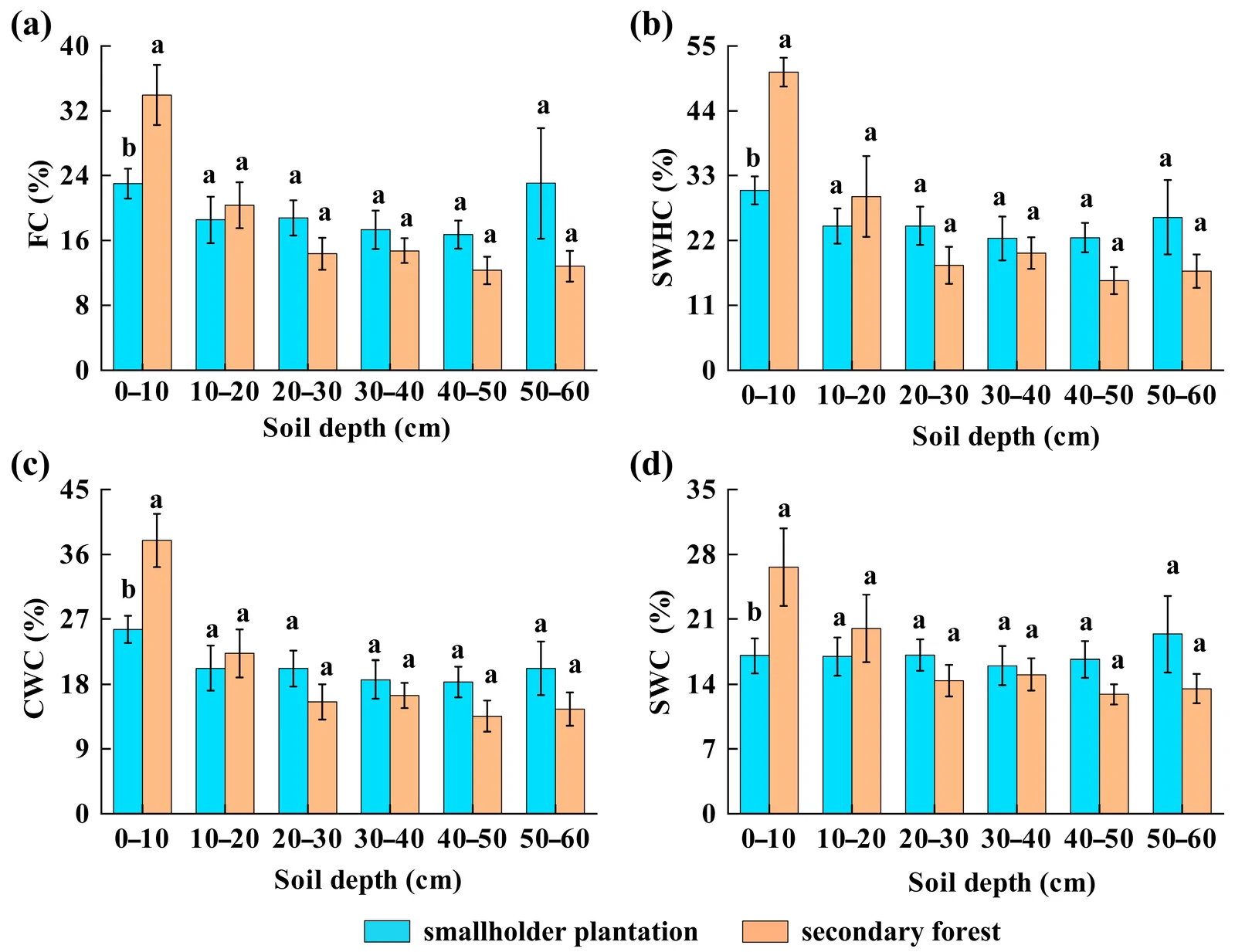
Soil water–holding capacity in plantation and secondary forests at different soil depths: (**a**) field capacity (FC), (**b**) soil water-holding capacity (SWHC), (**c**) capillary water capacity (CWC), and (**d**) soil available water content (SWC). Bars represent mean ± SE. Letters above bars are indicative only; effect sizes are reported in the text (effects reported as in 4.4; given *n* = 3 plots per type, these are indicative rather than confirmatory).

Water-holding properties (Figure 4) mirrored this pattern. All four indicators—field capacity (FC), soil water-holding capacity (SWHC), capillary water capacity (CWC), and soil available water content (SWC)—were consistently higher in secondary forests in the 0–10 cm layer (FC ≈ 34% vs. ≈22%; SWHC ≈ 50% vs. ≈24%; CWC ≈ 37% vs. ≈24%; SWC ≈ 27% vs. ≈16%); at 10–60 cm the two forest types shared letters at every depth for all indicators. Given *n* = 3 plots per forest type, surface contrasts are reported as effect sizes with their uncertainty ranges rather than strict significance thresholds (see Section 4.4).

Finally, we asked whether the four variables implicated in the proposed aboveground route co-vary along the expected sequence. Across the six plots, the four consecutive associations—understory H′ with litter mass, litter mass with SOM, SOM with non-capillary porosity, and non-capillary porosity with steady-state infiltration—were all positive (correlation coefficients 0.78, 0.82, 0.69 and 0.74), but none was statistically significant. Because six plot-level observations cannot support a path model, we report these associations descriptively as a co-variation sequence; they are neither fitted pathways nor evidence of mediation.

### 2.4. Root–Water Matching: Depth Alignment of Fine Roots and Soil Water Storage

To assess whether root distribution aligns with plant-available water, we evaluated fine-root biomass (FRB) and soil available water content (SWC) profiles (Table 2; layer-by-layer raw FRB and SWC profiles are given in Table S1). In the secondary forest, FRB was strongly concentrated in the 0–20 cm layer, accounting for 68.2 ± 4.5% of total root biomass. SWC followed a similar pattern, peaking at 18.2 ± 1.0% in the 0–10 cm layer. In contrast, the plantation exhibited a more evenly distributed root profile, with only 42.3 ± 3.8% of FRB in the 0–20 cm layer, while SWC peaked at 20–30 cm (15.3 ± 0.8%), where root density was sparse.

The root-water matching coefficient (MC) was 0.89 for the secondary forest, indicating relatively high spatial alignment, versus only 0.41 for the plantation (Table 2). Root-available water (RAW), expressed as an equivalent water depth, was 8.7 ± 0.6 mm in the secondary forest, nearly three times the 2.9 ± 0.3 mm of the plantation, a large effect size reported without a significance threshold given *n* = 3 plots per forest type (see Section 4.4).

## 3. Discussion

### 3.1. Canopy Interception Saturation and Trait-Associated Higher Stemflow

The ~500 mm annual precipitation divergence between the two study sites, likely influenced by local topographic and windward-slope effects [19,20], imposes recognized constraints on cross-site water-budget closure. We therefore refrain from over-interpreting absolute flux differences and rely on scale-invariant metrics—normalized partitioning percentages and DBH-normalized regression slopes—for trait-mediated hydrological regulation inference [10,21,22].

The canopy interception contrast we report is consistent with saturation dynamics: under low-intensity rainfall, interception efficiency is high, but during extreme tropical storms canopy storage capacity (S_canopy) saturates and diverts excess water to throughfall and stemflow [23,24]. Counter-intuitively, the wetter plantation still exhibited a substantially higher stand-level stemflow fraction (20.96%) than the secondary forest (7.18%); these stand-level values are partitioning percentages computed by aggregating stemflow volumes of ten representative trees per forest type and dividing by plot rainfall. This stand-scale, plot-area-normalized yield is not directly comparable with crown-projection-normalized stemflow percentages, which are typically < 5% in humid tropical forests; exceptionally high stemflow yields are nevertheless documented for smooth-barked, steep-branched plantation species [12,14,19]. Because this pattern is also contrary to the expectation that heavier rainfall saturates canopy storage and depresses stemflow proportion, it provides objective evidence that morphological traits (low-BRI smooth bark and large DBH) and stand composition, rather than absolute precipitation input, co-vary with canopy partitioning. Smooth-bark trunks act as low-resistance funnels that concentrate rainfall into localized stemflow hotspots around tree bases [21,25], consistent with the hydrodynamic principle that smooth surfaces minimize flow resistance along the stem exterior.

Late-successional native species (*Hopea hainanensis* and *Sindora glabra*) invest heavily in structural and defensive tissues, producing high-bark-roughness (BRI) and lignified fissures that retard stemflow rates and promote stem water absorption [12,25]. Their surface-concentrated fine-root biomass (4.8 ± 0.5 vs. 2.1 ± 0.3 t·ha^−1^ in the 0–10 cm layer; Table 2) plausibly modifies the soil matrix, fostering non-capillary porosity (NCP ~19%) that accommodates rapid infiltration [18]. Belowground, infiltration follows Green–Ampt non-linear threshold dynamics [26], where the transition from matrix absorption to surface runoff is governed by the ratio of rainfall intensity to soil saturated hydraulic conductivity (Ksat) [27,28]. In the plantations, topsoil structural degradation depresses Ksat, causing frequent threshold exceedance during high-intensity tropical storms [29,30]; localized stemflow funneling at stem bases may then exacerbate hydraulic overload and accelerate topsoil erosion around plantation trunks [31].

### 3.2. Functional Redundancy and Root Traits in Secondary Forests

The superior ecohydrological performance of naturally regenerated secondary forests is consistent with the functional-redundancy-driven ecosystem-insurance hypothesis [32]. Secondary forests assemble multi-species mixtures where co-occurring native taxa (*Hopea hainanensis*, *Sindora glabra*, *Dacrydium pectinatum*) exhibit complementary and partially overlapping hydrological traits [22,33,34]. Overlapping trait portfolios create functional redundancy: when individual species undergo seasonal leaf senescence or localized disturbance, co-existing taxa maintain canopy interception and kinetic energy dissipation, buffering ecosystem hydrology against typhoon-driven rainfall in Hainan [35].

This multi-tiered structural barrier (high-tier canopy, mid-tier shrubs, ground-level litter) progressively dissipates droplet energy [18]. Aboveground trait diversity propagates belowground via positive plant–soil feedbacks [29]. Multi-species communities supply chemically diverse litter alongside vertically stratified fine-root networks; we directly measured fine-root biomass (FRB), and FRB is more strongly concentrated in surface layers in secondary forests than in plantations (Section 2.4). Living roots generate micro-fissures, while decaying roots leave continuous channels that coalesce into macro-biopores [17,18]. These biopores are inferred to promote preferential flow, bypassing compacted soil matrices to accelerate deep infiltration [33].

Profile analysis demonstrates a critical dimension of root functional redundancy: secondary forests achieve tight spatial coupling between fine-root biomass peaks and soil available water content (SWC) in the top 0–20 cm rhizosphere (MC = 0.89). Conversely, the plantations exhibit hydrological decoupling (MC = 0.41), trapping plant-unavailable water in deep clay horizons where root density is minimal [36]. During dry spells, deep-rooting native species are inferred to perform hydraulic lift [37,38]; during intense storms, the interwoven root matrix acts as an underground drainage network, providing dual-directional hydrologic buffering [39].

### 3.3. Forest Restoration: Toward Disentangling Human Disturbance from Intrinsic Trait Effects

Restoring converted tropical landscapes requires shifting from simple canopy-cover targets toward nature-based solutions centered on plant functional trait optimization [22]. Traditional management prioritizes individual tree growth while ignoring multi-dimensional trait combinations required for integrated watershed regulation. Crucially, sustainable management must explicitly distinguish the negative impacts of anthropogenic practices (e.g., periodic understory weeding and litter clearing) from intrinsic species trait effects. In commercial plantations (*Hevea brasiliensis* and *Areca catechu*), litter clearing directly depletes SOM and disrupts macro-aggregate formation, exacerbating infiltration failure independently of canopy traits [29,40,41].

The diversity signal in our co-variation pattern should not be read as a direct causal effect of species richness per se. Understory Shannon H′ (2.8 ± 0.2 in secondary forest vs. 1.2 ± 0.1 in plantations) is more parsimoniously interpreted as a composite proxy for reduced anthropogenic disturbance: secondary forests experience no litter clearing or weeding, whereas plantations are subject to routine understory removal [42]. The cascade therefore represents a management-mediated feedback in which disturbance cessation is associated with greater litter accumulation, elevated SOM and NCP, and sustained infiltration, with trait complementarity acting as the proximate mechanism. At *n* = 6 this remains co-variation evidence rather than confirmatory mediation, consistent with the management–trait syndrome framing developed below.

Placing our two-type contrast within the broader land-use gradient available from the same regional inventory sharpens the management–trait syndrome framing. Cheng et al. [43] reported that the primary-to-secondary-forest transition alone is associated with substantial degradation in litter mass, soil water-holding capacity and 0–20 cm bulk density, with the secondary-to-plantation transition imposing an additional, qualitatively similar signal. Read alongside our own gradient, a substantial portion of the total soil-hydrological gap between rubber-dominated and secondary stands appears to be inherited from the primary-to-secondary transition (selective logging, edge effects, species turnover), with the remainder accumulating during the secondary-to-plantation transition (weeding, litter removal, single-trait dominance). This supports our framing: anthropogenic disturbance and trait simplification are not independent drivers but co-vary along the same land-use axis, and any restoration that targets only one dimension (e.g., replanting native species without ceasing weeding) is unlikely to recover pre-disturbance hydrological function.

To rehabilitate ecohydrological function in degraded plantations, management should transition from simple density enrichment to a ‘Trait-Synergy’ restoration framework [44]: targeted enrichment planting of native, functionally complementary backbone species (e.g., *Hopea hainanensis*), with selection criteria coupling aboveground stemflow-retardation traits (high BRI and inferred high BWHC) with belowground complementary fine-root architecture (surface FRB accumulation, the directly measured trait here). Enrichment planting must be paired with reduced weeding intensity to allow understory PFTs to establish [18,31].

Two complementary operational adjustments extend this Trait-Synergy framework beyond species choice. First, understory weeding and litter-raking should be eliminated or restricted to the first 2–3 years post-establishment, allowing litter-derived plant–soil feedbacks to develop before management intervention. Second, restoration targets should be expressed as the candidate trait-based indicators developed in Section 3.4 (understory H′ ≥ 1.5; BRI ≥ 2.0; MC ≥ 0.80) rather than canopy-cover percentage alone, and should be paired with a suggested management target—weeding frequency ≤ 1 yr^−1^ beyond the establishment phase—so that trait and management are pursued jointly. We cite Shannon H′ strictly as the understory diversity index actually measured here; functional-diversity indices such as FRic, FEve, FDiv and Rao’s Q were not computed in this dataset.

Field validation of these trait-based candidate restoration indicators in factorial experiments is the necessary next step before the proposed management–trait syndrome framework can be operationally adopted in tropical smallholder landscapes. More broadly, species-based rehabilitation of degraded land through deliberate planting combinations has also been advanced in temperate systems—for example, herb–legume interplanting for sandy-land recovery [45]—which parallels our call for validating trait-based candidate indicators across contrasting restoration contexts.

### 3.4. Limitations and Future Directions

Our study has five main constraints, each of which limits causal inference but also points to concrete future work. Small replication and statistical power. With three plots per forest type (*n* = 3 vs. 3; total *n* = 6), power to detect Cohen’s d = 1 is only ~16%, and all bootstrap 95% CIs crossed zero. We therefore report effect sizes rather than significance thresholds and treat the path model as hypothesis-generating. This constraint defines the study’s main contribution: a multi-indicator paired baseline from a real managed landscape, with directional effect sizes (e.g., ~3-fold stemflow fraction, ~16-fold lower steady-state infiltration, root–water matching 0.89 vs. 0.41) that can inform pre-registration and sample-size planning. Future work should increase replication to 8–10 plots per type across multiple catchments and use mixed-effects models to formally test the proposed pathways. Management × trait confounding. Routine weeding and litter removal in plantations are intrinsically confounded with species trait effects. We frame this as a “management–trait syndrome” rather than pure trait effects—an ecological reality in smallholder landscapes. The study contributes by identifying measurable trait indicators (bark roughness, surface fine-root biomass, root–water matching) that can be paired with management metrics in future factorial experiments crossing weeding frequency with species mixtures, with “management intensity” included as an explicit variable. Site and rainfall confounding. The two sites differ by ~500 mm annual rainfall and are within a single ~10 km^2^ area, so spatial and forest-type effects are partially confounded. We used scale-invariant metrics (normalized percentages, DBH-normalized slopes, bootstrap CIs), and a ~16-fold infiltration difference is unlikely to be fully explained by a ~1.24-fold rainfall difference; still, residual rainfall and topographic effects cannot be excluded. Likewise, several mechanistic explanations invoked in the Discussion—preferential flow, macropore development, hydraulic lift, deep soil moisture storage, and stem-base erosion—are interpretive hypotheses consistent with the observed co-variation rather than directly measured processes. Future work should replicate the paired design across independent catchments along isohyets and test for spatial autocorrelation. Stemflow upscaling and DBH representation. Stemflow upscaling rests on the ten instrumented trees per forest type drawn from the twelve-tree structural survey (Table S3): the surveyed trees span 3.5–33.5 cm DBH in the secondary forest and 13.2–25.7 cm in the plantation and were allocated across three DBH classes, but this is a structural subsample rather than a complete individual-tree inventory and the largest DBH class contains only two (secondary forest) to five (plantation) trees, so residual selectivity cannot be fully excluded. Infiltrometer insertion artefact. Hammer-driven ring insertion can compact the ring wall and smear pores, biasing absolute rates downward. Because the same procedure was used in both forest types, and the well-aggregated secondary-forest soil is more susceptible to compaction, this artefact would compress rather than inflate the between-forest difference; the ~16-fold contrast is therefore a conservative lower bound. Undisturbed-core non-capillary porosity agrees directionally, offering independent corroboration. Future work should pair double-ring measurements with tension infiltrometers or undisturbed-core estimates. Implications for restoration and management. Despite these limitations, the exploratory patterns have practical relevance. The proposed trait-based indicators—understory Shannon H′ ≥ 1.5, bark roughness index BRI ≥ 2.0, and root–water matching coefficient MC ≥ 0.80—are candidate restoration indicators for future validation beyond canopy cover alone. They are illustrative, not statistically derived thresholds, and need field validation. Reducing understory weeding and retaining litter may help rebuild soil organic matter, non-capillary porosity, and infiltration, while enrichment planting with native, functionally complementary species (e.g., *Hopea hainanensis*) may enhance stemflow retardation and surface fine-root accumulation. These suggestions should be tested in long-term controlled trials. The study’s value lies in providing (i) a multi-indicator paired baseline, (ii) a reusable root–water matching metric, and (iii) a management–trait syndrome framework that translates exploratory co-variation into testable hypotheses and candidate restoration indicators.

Therefore, we position this work explicitly as an exploratory, paired baseline at plot scale rather than as a confirmatory test of the proposed pathway. Its principal contribution is the simultaneous measurement of four under-characterised trait–soil–hydrology links in a real tropical smallholder landscape, plus the reusable root–water matching metric and the candidate (illustrative, not statistically derived) restoration thresholds (H′ ≥ 1.5; BRI ≥ 2.0; MC ≥ 0.80). Validation of these indicators in factorial experiments that cross weeding frequency with species mixtures—ideally coupled to isotope tracing and N:P stoichiometry—remains the necessary next step before the proposed candidate indicators can be considered for operational adoption.

## 4. Materials and Methods

### 4.1. Study Area

The study was conducted in Qixianling National Forest Park (18°39′–18°48′ N, 109°34′–109°42′ E), Baoting Li and Miao Autonomous County, southern Hainan, China (Figure S1). The site lies in the tropical monsoon climate zone (mean annual temperature 23–25 °C; long-term mean annual precipitation 1900–2600 mm, with > 80% in the wet season May–October); during the 2023 study year, site-level rainfall totalled 2571.7 mm at the plantation and 2074.9 mm at the secondary-forest site. Soils are granite/sandstone-derived Ferralic Acrisols and primitive Cambisols [46]; more detailed site characterization, including elevation, slope and soil physico-chemical profile, is given in the Supplementary Materials.

Two 15–20-year-old forest types were selected on matched slope, aspect, elevation and parent material following the comparative design framework of Chen et al. [47]: (i) a mixed smallholder plantation of *Hevea brasiliensis* (rubber tree) and *Areca catechu* (betel nut palm) at an approximately 6:4 stem-number ratio, under routine understory weeding (2–3 times yr^−1^) and litter removal—the dominant land-use type in central Hainan since the 1990s [48], and (ii) secondary forest, naturally regenerated evergreen broadleaved forest recovering from selective logging, dominated by *Hopea hainanensis*, *Sindora glabra* and *Dacrydium pectinatum*. Both types occur within a 2 km radius and shared the same regional climate, soil type (Ferralic Acrisols and primitive Cambisols [46]) and disturbance history until the plantation was established ~15–20 years before sampling; they differ, however, in local topographic and windward exposure, which underlies the ~500 mm difference in site-level rainfall recorded during the 2023 observation year (Section 3.4). Stand structural attributes (age, canopy density, DBH, height, crown width, basal area) for the three replicate plots per type are summarized in Table S2; canopy density (40 ± 2% vs. 60 ± 3%) and mean DBH (19.6 ± 1.2 cm vs. 12.3 ± 0.9 cm) confirm the trait simplification we document. Because both planted species share identical silvicultural protocols, the dominant stand-scale signal is management × trait rather than species identity; we frame the contrast as a smallholder-management baseline (Section 3.4).

### 4.2. Plot Design, Vegetation Survey and Field Measurements

To ensure the representativeness and replicability of hydrological observations, three replicate 20 × 20 m main plots were established within each forest type, and each main plot was subdivided into three 10 × 10 m sampling subplots (the remaining area was left as a buffer). The plot, not the subplot, rain gauge or soil core, is the independent experimental unit (*n* = 3 plots per type); all subsamples were spatially nested within plots and later aggregated to plot level before inferential testing (4.4). Stand structural attributes are summarized in Table S2.

Rainfall partitioning. Bulk rainfall (P) was measured in an adjacent open clearing by a tipping-bucket rain gauge (HOBO U30, Onset Computer Corporation, Bourne, MA, USA) installed 150 m from the plots and raised 20 cm above ground. Throughfall (TF) was measured with five calibrated tipping-bucket gauges per plot (0.1 mm tip^−1^), installed 1.0–1.5 m above ground to avoid understory obstruction. Stemflow (SF) was collected from ten representative trees per forest type stratified across three DBH classes and, because the plantation is a mixed rubber–betel nut stand (~6:4 stem-number ratio), selected across both species in proportion to that ratio, using spiral-hose systems attached at 1.3 m and angled at 30° from vertical to channel water into calibrated collectors. Plot-level stemflow was computed by DBH-class weighting (Equation (1)) and expressed as an equivalent water depth over the entire plot area; the yield was therefore not normalized by crown projection area, so it represents stand-scale stemflow input rather than a per-unit-crown-area flux. Of the twelve trees per forest type characterised in the structural survey (Table S3), ten were instrumented for stemflow and allocated across the three DBH classes in proportion to each class’s share of the plot inventory; the surveyed trees span 3.5–33.5 cm DBH in the secondary forest and 13.2–25.7 cm in the plantation (mean ± SE, 12.28 ± 2.52 cm and 19.57 ± 1.40 cm, respectively), with 5/5/2 and 2/5/5 trees in the low, middle and high DBH class. Plot-level stemflow was upscaled by within-class DBH weighting rather than by a single stand-mean tree. Canopy interception (CI) was derived from the water balance CI = P − TF − SF (Equation (2)). Throughfall uniformity (TU) was quantified as 1 minus the spatial coefficient of variation among gauges (Equation (3)).

(1) $$\mathrm{SF}=\sum_{i=1}^{N}\frac{\mathrm{Ci}\;\times \;\mathrm{Mi}}{S\;\times 1000}\;$$

where SF represents the stemflow in the plot (mm), N is the number of tree diameter classes, Ci is the average stemflow volume of class i (mL), Mi is the number of trees in class i, and S is the plot area (m^2^).

*CI* = *P* − *TF* − *SF* (2)

where CI is the canopy interception (mm), P is the rainfall measured outside the forest (mm), TF is the throughfall recorded within the forest (mm), and SF is the stemflow measured (mm).

*TU* = 1 − *CV* (3)

where CV is the spatial coefficient of variation of throughfall among gauges, calculated as:

$$\mathrm{CV}=\frac{\mathrm{SD}}{\bar{x}}$$

Undisturbed soil cores (100 cm^3^ ring knife) were collected at 0–10, 10–20, 20–30, 30–40, 40–50 and 50–60 cm depths from five random points per subplot (*n* = 45 per forest type per depth). Soil total C and N were determined with an elemental analyzer (Elementar Vario EL III, Langenselbold, Germany), SOM by potassium dichromate oxidation, and available N and P with a continuous flow analyzer (SKALAR, Breda, The Netherlands). Particle size distribution was measured with a Malvern Mastersizer 2000 laser analyzer (Malvern, UK).

Soil infiltration was measured in situ with a double-ring infiltrometer (TI01–TURF–TEC; inner/outer rings 10/20 cm diameter, Turf-Tec International, Tallahassee, FL, USA). Rings were driven to 30 cm depth by hammering, using an identical procedure in both forest types; the deep insertion, together with the outer buffer ring, was intended to suppress lateral flow and approximate one-dimensional infiltration. Steady-state infiltration and saturated hydraulic conductivity were derived following [49]. Because hammer-driven insertion can compact the ring wall, smear pore openings and disrupt macropore continuity, absolute rates reported here should be regarded as conservative; the bearing of this artefact on the between-forest contrast is evaluated in Section 3.4.

Understory plant diversity. Understory vegetation (shrubs and herbs) was surveyed in five quadrats per plot; species richness and relative abundance were used to compute the Shannon–Wiener index (H′; Equation (4)). Throughout the text, “plant diversity” refers to this understory H′ and not to tree diversity.

(4) $$H^{\prime }=-\sum_{i=1}^{S}p_{i}\ln p_{i}$$

Soil water-holding capacity. Bulk density (BD), soil water-holding capacity (SWHC), capillary water-holding capacity (CWC), field capacity (FC), and soil water storage (SWS) were derived from standard ring-cutter weighing at four sequential saturation/drainage/oven-drying steps following standard gravimetric ring-cutter procedures [50].BD is bulk density (g/cm^3^), SWHC soil water-holding capacity (%), CWC capillary water-holding capacity (%), FC field capacity (%), and SWS soil water storage (mm) computed for the natural, field, capillary and saturated states, respectively; V is core volume (cm^3^), M the ring-cutter mass without caps (g), M_1_–M_4_ the masses of the ring cutter plus soil after saturation, capillary saturation, field-capacity drainage, and oven-drying, respectively (g), h soil thickness (cm), and θ gravimetric water content (%). Total porosity (TP), capillary porosity (CP) and non-capillary porosity (NCP) follow [50]:

*BD* = (*M*4 − *M*)/*V* (5)

*SWHC* = (*M*1 − *M*4)/(*M*4 − *M*) × 100% (6)

*CWC* = (*M*2 − *M*4)/(*M*4 − *M*) × 100% (7)

*FC* = (*M*3 − *M*)/(*M*4 − *M*) × 100% (8)

*SWS* = *h* × *BD* × *θ* × 10^−1^ (9)

*TP* = (1 − *BD*/*ds*) × 100% (10)

(11) CP = CWC × BD

*NCP* = *TP* − *CP* (12)

where TP is the total porosity (%), ds is the soil particle density (assumed to be 2.65 g/cm^3^), CP is the capillary porosity (%), and NCP is the non-capillary porosity (%). Vegetation characteristics (species composition, stand age, DBH, canopy density, crown width) and soil properties were surveyed monthly throughout 2023 (Table S2).

### 4.3. Plant Functional Trait Measurements

Variables reported below are explicitly classified as directly measured functional traits, stand-structural variables, soil variables, management variables, or literature-derived/contextual traits (the latter used only qualitatively). Leaf area index. LAI was measured in situ during the peak growing season (July–August) with an LAI-2200C canopy analyser (LI-COR, Lincoln, NE, USA). Fifteen systematic sampling points were established per plot under diffuse light; one above-canopy reference reading and four below-canopy replicates were recorded per point, and plot-averaged effective LAI was derived with FV-2200 software.

Bark roughness index (BRI). BRI was measured at breast height (1.3 m) on the north-facing trunk surface of ten representative trees per forest type using a comb-type bark-profile gauge with multiple pins over a 100 mm span. BRI was defined as the standard deviation of pin displacements (Equation (13)):

(13) $$\mathrm{BRI}=\sqrt{\frac{1}{n-1}\sum_{i=1}^{n}{\left(x_{i}-\bar{x}\right)}^{2}}$$

Root–water spatial matching coefficient (MC). To assess whether fine-root distribution aligns with plant-available water across the soil profile, the spatial matching coefficient between fine-root biomass (FRB) and soil available water content (SWC) was computed as the depth-weighted overlap of the two normalized profiles (Equation (14)): root-available water (RAW) was obtained by depth-integrating the plant-available water content over the soil horizon occupied by fine roots and is reported as an equivalent water depth (mm; 1 mm = 10 t·ha^−1^).

(14) $$\mathrm{MC}=1-\frac{1}{2}\sum_{i=1}^{L}|r_{i}-w_{i}|$$

### 4.4. Statistical Analysis

(1) Plot-level inference. All analyses were performed in R 4.2.1 [51], with lme4 1.1-31, emmeans 1.8.5, vegan 2.6-4, randomForest 4.7-1.1 and pwr 1.3-0. Because measurement points, subplots and gauges were nested within three replicate plots per forest type, the plot was the sole independent experimental unit; subsamples were averaged to subplot and then to plot before testing. Plot-level variables (canopy density, DBH, BRI, LAI, litter stock, understory H′) thus form a two-group comparison with *n* = 3 per type (residual df = 4; ≈16% power to detect Cohen’s d = 1 at α = 0.05, from pwr.t.test two-sided *n* = 3 vs. 3); we therefore report effect sizes and 95% CIs and refrain from over-interpreting significance thresholds. Depth-resolved soil variables were analyzed with mixed-effects models (lme4::lmer [52]) as value ~ forest_type × depth, with plot and subplot nested within plot as random effects (1 | plot/subplot); fixed effects were tested by type-III Wald F-tests with Kenward–Roger df, and contrasts estimated by emmeans with Tukey adjustment. DBH–stemflow slopes were compared by ANCOVA with forest type as the factor and DBH as the covariate.

(2) Multivariate coupling. Bivariate associations among soil physical and hydrological properties are summarized by Pearson correlation matrices (Figure S5). Redundancy analysis (RDA, vegan::rda [53]; Figure S6) related soil hydrological responses (SWC, NCP, steady-state infiltration) to vegetation and soil predictors (canopy density, DBH, BRI, litter stock, understory H′, SOM, clay content); constrained-axis significance was tested by 999 permutations stratified by plot. Predictors with variance inflation factor (VIF) > 5 were removed; among water-retention variables only soil available water content (SWC) was retained, because SWHC, CWC and FC are algebraic transformations of the same ring-cutter weights. Because these analyses use plot-level means (*n* = 6), the RDA is exploratory and its explained-variance proportions are reported as a descriptive association map only. As a complementary, purely exploratory ranking of predictor importance for the three rainfall-partitioning fluxes, we additionally fitted random-forest models (R package randomForest) on the same vegetation and soil predictor set; variable-importance scores are reported descriptively only, without formal inference, in Figure S7. Both multivariate exercises—the RDA ordination and the random-forest screening—are presented as exploratory and predictive screening of candidate drivers, to be validated in larger factorial experiments rather than as fitted predictive models or operational restoration targets.

## 5. Conclusions

This study jointly quantified canopy stemflow, bark functional traits, and fine-root architecture in paired plantation and secondary forests in Hainan, China, and showed that the divergent eco-hydrological regimes between the two forest types are best framed as a coupled management–trait syndrome rather than as isolated species effects. The stand-scale bark-trait–stemflow co-variation was directly supported; the root-architecture and diversity–infiltration pathways were consistent with our predictions but only partially testable with the present design because preferential flow was not directly measured and the four consecutive associations linking understory diversity to infiltration were not significant at *n* = 6. We therefore position this work as an exploratory, paired baseline at plot scale that (i) provides a multi-indicator reference dataset coupling four under-characterised trait–soil–hydrology links in a real tropical smallholder landscape, (ii) introduces a reusable root–water matching coefficient as a quantifiable restoration indicator, and (iii) frames candidate—illustrative, not statistically derived—thresholds (H′ ≥ 1.5; BRI ≥ 2.0; MC ≥ 0.80) as candidate indicators for future validation. Validation of these indicators requires factorial, long-term manipulative designs that cross weeding frequency with species mixtures, ideally coupled to isotope tracing and explicit N:P stoichiometry, to extend the current water-partitioning baseline into the full Plant–Water–Nutrient programme of this Special Issue. Because the two forest types co-vary in both vegetation community and management context, the reported eco-hydrological divergence should be read as a joint vegetation–management outcome rather than a pure trait effect.

## Figures and Tables

**Table 1:** Annual Rainfall Partitioning Between Plantations and Secondary Forests. Abbreviations: P, precipitation; TF, throughfall; SF, stemflow; CI, canopy interception.

Forest Type	P (mm)	TF (mm) ± SE	TF (%) ± SE	SF (mm) ± SE	SF (%) ± SE	CI (mm) ± SE	CI (%) ± SE
Plantation	2571.7	1528.5 ± 28.01	59.45 ± 2.15	538.9 ± 17.93	20.96 ± 2.45	504.3 ± 4.28	19.61 ± 3.67
Secondary forest	2074.9	1241.2 ± 34.78	59.81 ± 4.46	149.0 ± 3.03	7.18 ± 2.95	684.7 ± 15.33	33.00 ± 5.24

**Table 2:** Fine-root distribution, soil available water content, and spatial matching index summary (Mean ± SE). Change magnitudes are the percentage by which the secondary forest exceeds the plantation. Within this table, ‘Proportion of FRB in 0–20 cm’ is computed per plot as the FRB summed over the 0–10 and 10–20 cm layers divided by the total FRB summed over the full sampled 0–60 cm profile, then averaged across plots (mean of per-plot proportions ± SE); it is therefore not identical to a ratio formed from profile-mean FRB values. RAW is reported as an equivalent water depth (mm; 1 mm = 10 t·ha^−1^; see Section 4.3).

Indicator	Secondary Forest	Plantation	Change Magnitude
Proportion of FRB in 0–20 cm layer (%)	68.2 ± 4.5	42.3 ± 3.8	+61.2%
Surface layer (0–10 cm) FRB (t·ha^−1^)	4.8 ± 0.5	2.1 ± 0.3	+128.6%
Peak SWC depth (cm)	0–10	20–30	—
Root-water matching coefficient	0.89	0.41	+117.1%
Root-available water (mm)	8.7 ± 0.6	2.9 ± 0.3	+200.0%

## Data Availability

The plot-level data supporting this study (rainfall partitioning, soil physicochemical properties, infiltration, fine-root biomass, soil water content, and derived indices) are provided in the Supplementary Materials (Tables S1–S3). The R code used for the mixed-effects models, ANCOVA, bootstrap sensitivity checks, and redundancy analysis is available from the corresponding author upon reasonable request. The datasets generated and analyzed during the current study are not publicly available but are available from the corresponding author upon reasonable request. Requests should be addressed to Pei Wang (peiwang@bnu.edu.cn).

## Supplementary Materials

The following supporting information can be downloaded at: https://www.mdpi.com/article/10.3390/plants15213307/s1, Figure S1: Study area and plot location maps; Figure S2: 0–60 cm full-depth soil particle composition bar charts + box plots (main text reports 0–60 cm profile key differences); Figure S3: Soil physical and chemical properties full-depth column charts (BD, TP, NCP, SOM, nutrients; main text highlights 0–60 cm results). Note that the 0–10 cm surface contrast (higher bulk density and lower total porosity in the plantation) is opposite in sign to the depth-weighted 0–60 cm profile means given in Section 2.3, where the plantation has the lower bulk density; the two are not interchangeable and should not be read as the same quantity; Figure S4: Cumulative soil water storage across the 0–60 cm soil profile, partitioned by depth—FS/SWHS/CWS/SWS; Figure S5: Pearson correlation matrix of soil physical and hydrological properties; Figure S6: Redundancy analysis (RDA) ordination diagram; Figure S7: Random-forest variable importance for canopy interception, throughfall and stemflow; Table S1: Raw profile data table for FRB and SWC across soil layers; Table S2: Stand structural characteristics table; Table S3: Structural characteristics of the 12 sampled trees per forest type—DBH, tree height and crown projection area; Table 2 (main text) reports the root–water spatial matching coefficient (MC).

## Abbreviations

The following abbreviations are used in this manuscript:

ANCOVA	Analysis of covariance
BD	Bulk density
BRI	Bark roughness index
BWHC	Bark water-holding capacity
CI	Canopy interception
CP	Capillary porosity
CV	Coefficient of variation
CWC	Capillary water-holding capacity
CWS	Capillary water storage
DBH	Diameter at breast height
FC	Field capacity
FRB	Fine-root biomass
FS	Field-capacity storage
HSD	Honestly significant difference (Tukey’s HSD)
LAI	Leaf area index
LES	Leaf Economics Spectrum
MC	Root–water matching coefficient
NCP	Non-capillary porosity
PFTs	Plant functional traits
RAW	Root-available water
RDA	Redundancy analysis
RES	Root Economics Space
RLD	Root length density
SD	Standard deviation
SE	Standard error
SF	Stemflow
SOM	Soil organic matter
SRL	Specific root length
SWC	Soil available water content
SWHC	Soil water-holding capacity
SWHS	Soil water-holding storage
SWS	Soil water storage
TF	Throughfall
TP	Total porosity
TU	Throughfall uniformity
