# Supplementary material for: Plant Functional Traits Are Associated with Canopy-to-Soil Hydrological Regulation in Tropical Forests: Evidence from Smallholder Plantations and Secondary Forests
Source: Plants (Basel). 2026 Oct 26;15(21):3307. doi: 10.3390/plants15213307 (PMC13765789; doi:10.3390/plants15213307)
Supplement: Supplementary file 1 [file plants-15-03307-s001.zip › plants-4626884-supplementary.pdf]

Supplementary Materials

(Note: Per formatting requirements, all detailed supporting profiles, correlations, and large data tables have been moved to Supplementary Materials S1–S4. Text summaries are provided in the main text; see Supplementary Materials for full tabular outputs and figures).

The following supporting information can be downloaded at:

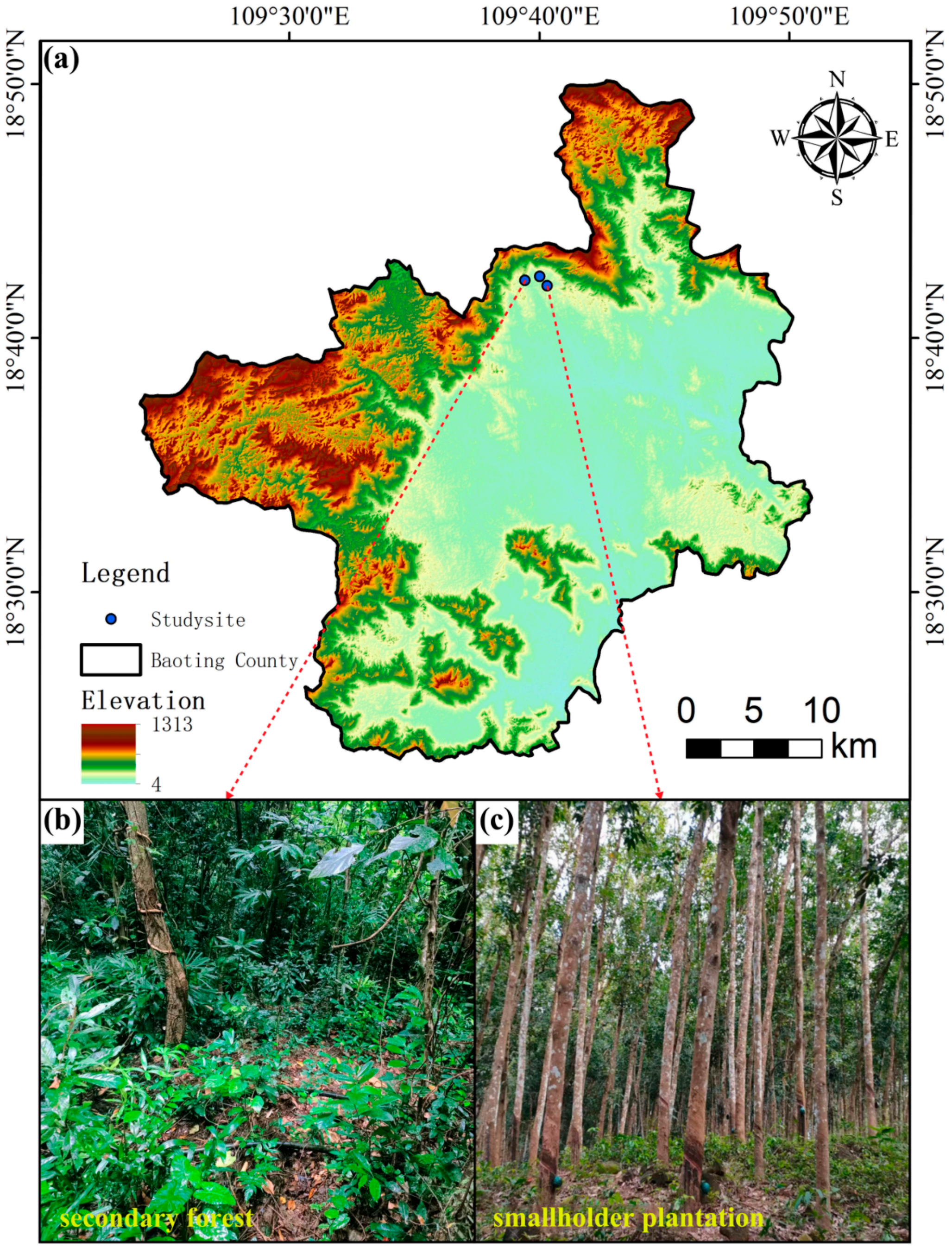

Figure S1. Geographic location of the study site at Qixianling National Forest Park, Hainan Island, China. (a) Elevation map showing study plot locations (blue dots) within Baoting Li

and Miao Autonomous County; (b) field photograph of secondary forest showing multi-layer canopy and diverse understory vegetation; (c) field photograph of the mixed rubber–betel nut smallholder plantation displaying uniform tree spacing and sparse understory.

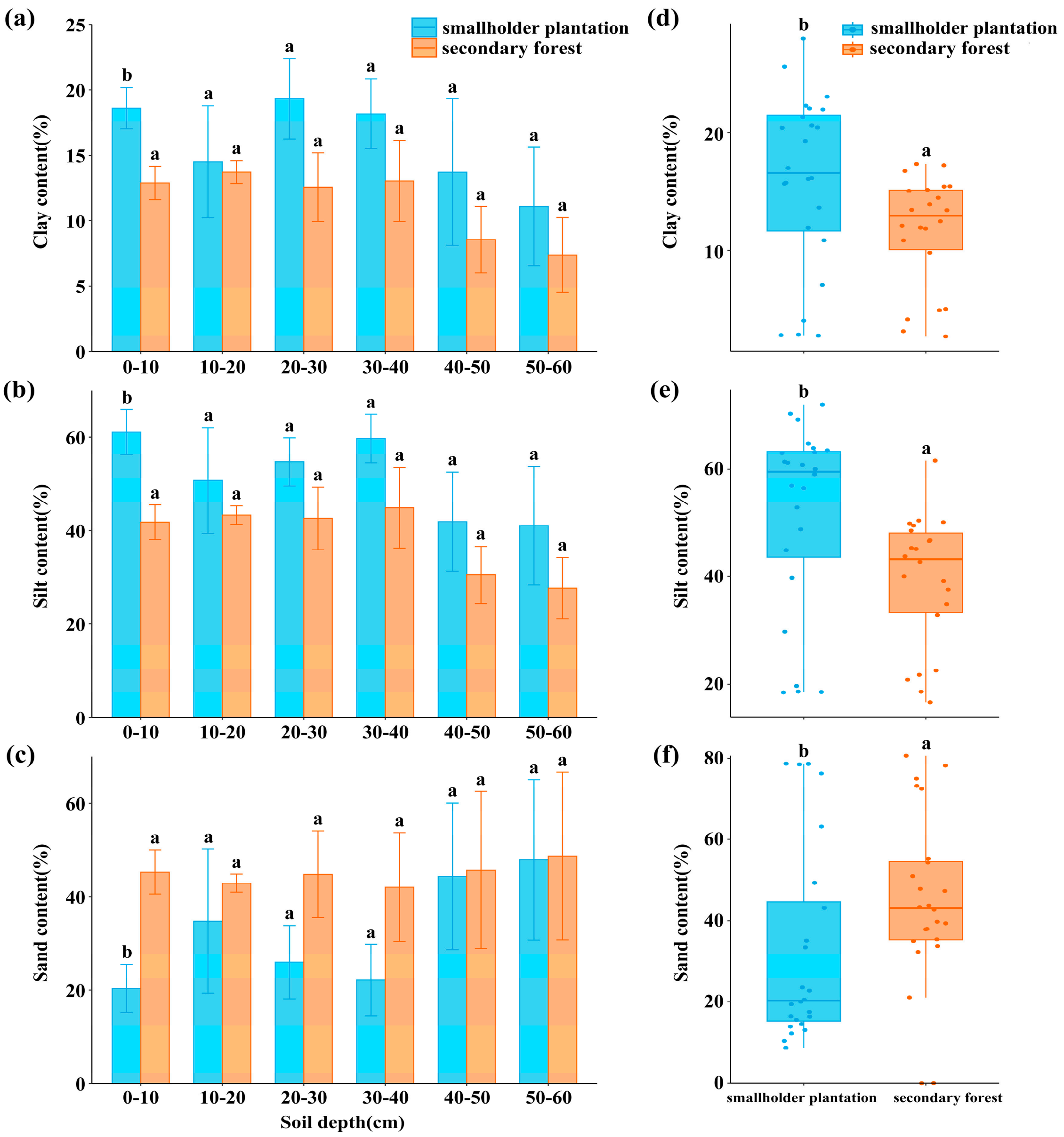

Figure S2. Soil particle-size distribution across 0–60 cm soil depth in smallholder plantation and secondary forests. Panels (a–c): depth-specific mean ± SE for clay, silt, and sand content; panels (d–f): box-and-whisker plots summarizing overall distribution of clay, silt and sand across all sampled depths. Different lowercase letters denote significant differences between forest types within identical soil depth ($p < 0.05$).

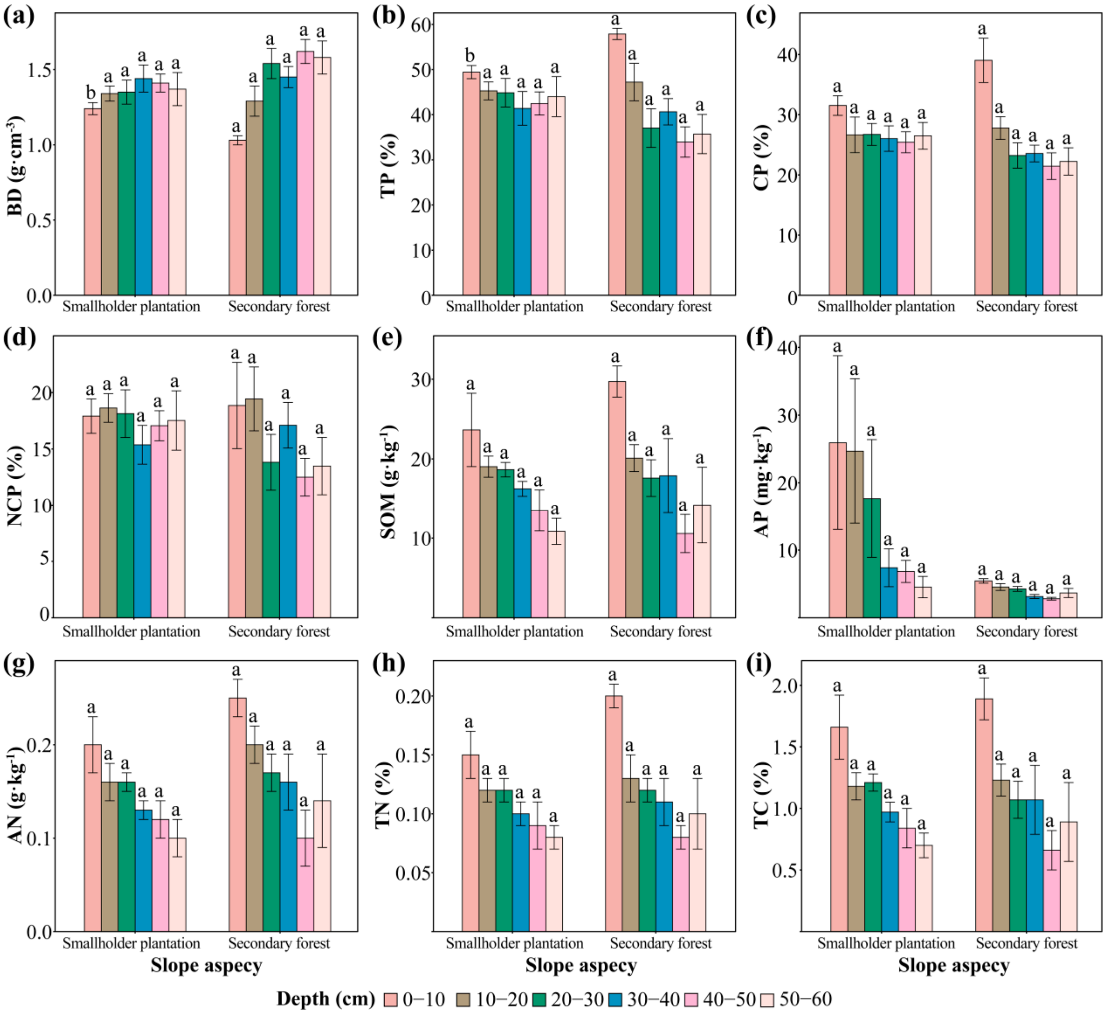

Figure S3. Soil physical and chemical properties along 0–60 cm soil profiles for smallholder plantation and secondary forests. Variables include bulk density (BD), total porosity (TP), capillary porosity (CP), non-capillary porosity (NCP), soil organic matter (SOM), available phosphorus (AP), available nitrogen (AN), total nitrogen (TN), and total carbon (TC). Error bars represent standard error of the mean (SE). Different lowercase letters indicate significant differences between forest types at each corresponding soil depth (p < 0.05).

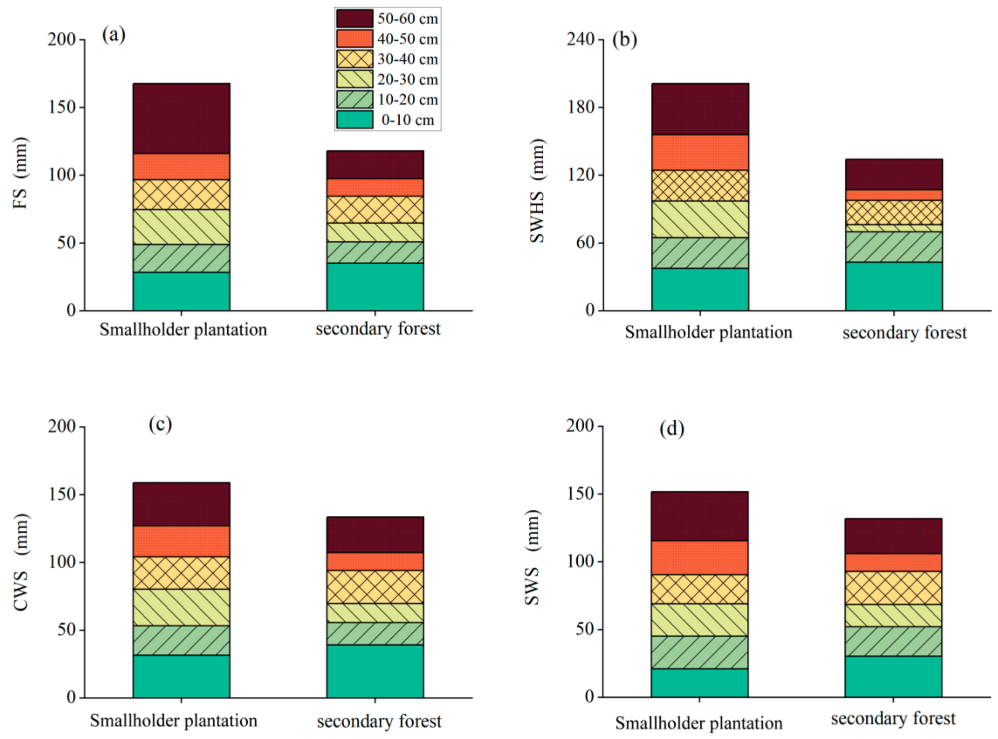

Figure S4. Cumulative soil water storage across the complete 0–60 cm soil profile for smallholder plantations and secondary forests. Stacked bars partition total storage by discrete depth intervals (0–10, 10–20, 20–30, 30–40, 40–50, and 50–60 cm). (a) Field capacity storage (FS); (b) soil water-holding storage (SWHS); (c) capillary water storage (CWS); (d) soil available water storage (SWS).

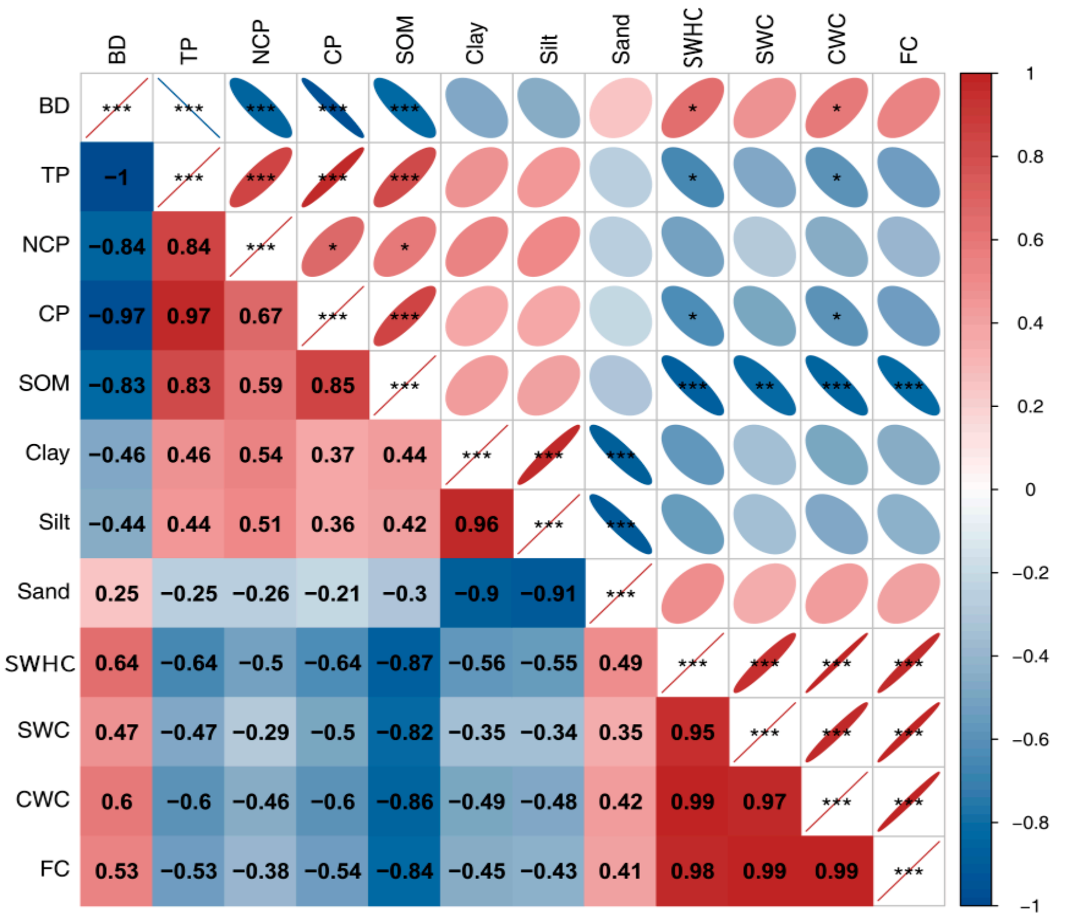

Figure S5. Pearson correlation matrix of measured soil physical and hydrological properties pooled across smallholder plantation and secondary forest plots. Ellipse orientation and color indicate correlation direction and magnitude. Asterisks mark significance levels: * *p* < 0.05; ** *p* < 0.01; * *p* < 0.001. BD, bulk density; TP, total porosity; NCP, non-capillary porosity; CP, capillary porosity; SOM, soil organic matter; SWHC, soil water-holding capacity; SWC, soil available water content; CWC, capillary water capacity; FC, field capacity.**

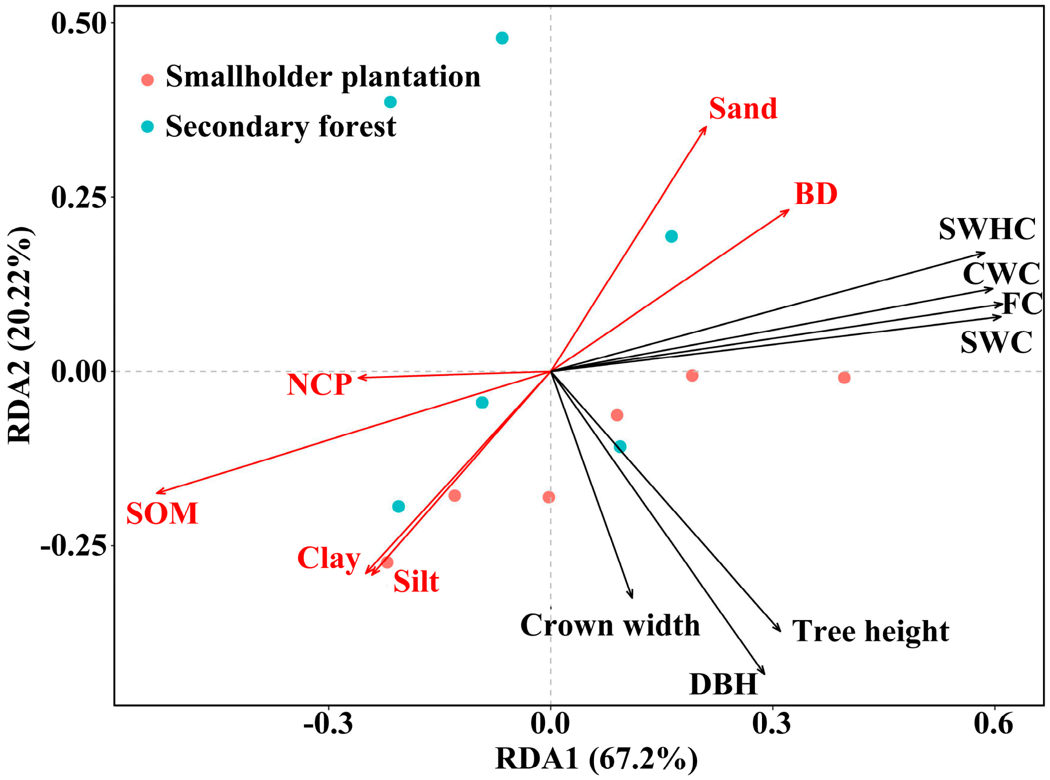

Figure S6. Redundancy analysis (RDA) ordination diagram illustrating associations among soil physical properties (red vectors), tree structural attributes (black vectors), and soil hydrological indicators. Blue circles denote secondary forest samples; orange circles denote plantation samples. Axes RDA1 and RDA2 explain 67.2% and 20.2% of total constrained hydrological variance, respectively.

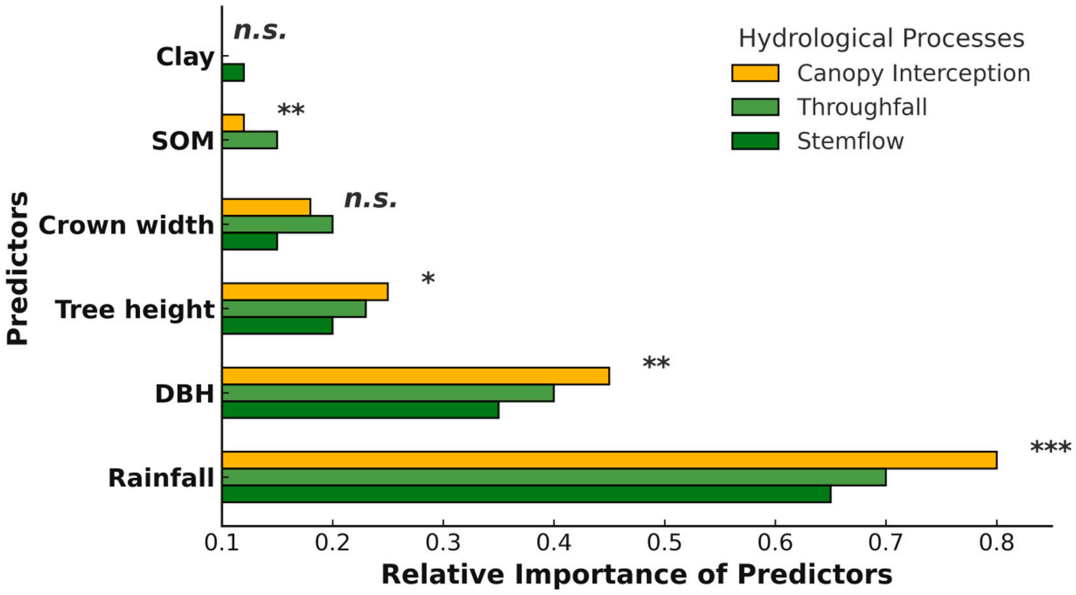

Figure S7. Relative importance of key predictors influencing canopy interception, throughfall, and stemflow across the studied forest stands. Bars indicate variable contributions derived from the random forest model, and colours distinguish hydrological processes (yellow for canopy interception, green for throughfall, and dark green for stemflow). Error bars represent model variance. Asterisks denote levels of significance (*$p < 0.05$, ** $p < 0.01$, *** $p < 0.001$; n.s., not significant). The absence of gridlines and the use of enhanced contrast ensure clarity for print and online publication.

Table S1. Fine-root biomass (FRB) and soil water content (SWC) along sequential soil profiles (5–55 cm depth intervals) for secondary forests and smallholder plantations. Values are mean values. FRB, fine-root biomass; SWC, soil water content. Units: FRB ($g \cdot dm^{-3}$); SWC (%).

Depth (cm)	Secondary forest FRB ($g \cdot dm^{-3}$)	Secondary forest SWC (%)	Plantation FRB ($g \cdot dm^{-3}$)	Plantation SWC (%)
5	4.8	18.2	2.1	14.9
15	3.2	15.5	1.5	14.5
25	1.5	13.2	1.8	15.3
35	0.8	11.8	1.2	15.0
45	0.4	10.5	0.9	14.2

Depth (cm)	Secondary forest FRB (g·dm⁻³)	Secondary forest SWC (%)	Plantation FRB (g·dm⁻³)	Plantation SWC (%)
55	0.2	9.8	0.6	13.5

Note: FRB, fine-root biomass; SWC, soil water content

Table S2. Stand structural and community characteristics of smallholder plantations and secondary forests. DBH, diameter at breast height; values are presented as mean ± SE. n (plots) indicates number of replicated experimental units. Here ± SE is the between-plot standard error (n = 3 plots); the tree-level SE of the 12 surveyed trees is given in Table S3.

Forest type	n(plots)	Stand age(yr)	Canopy density(%)	Mean DBH(cm) ± SE	Mean height(m) ± SE	Mean Crown width(m^2) ± SE	Basal area (m^2 ha^{-1}) ± SE
Plantation	3 (9 subplots)	15–20	40 ± 2	19.6 ± 1.2	9.4 ± 0.5	1.84 ± 0.08	34.5 ± 1.2
Secondary forest	3 (9 subplots)	15–20	60 ± 3	12.3 ± 0.9	5.2 ± 0.3	1.59 ± 0.07	29.8 ± 1.0

Table S3. Structural characteristics of the sampled trees (12 per forest type): diameter at breast height (DBH), tree height and crown projection area. Rows 1–12 are individual trees; the last three rows give mean ± standard error (SE), standard deviation (SD) and range. The sampled trees span 3.5–33.5 cm DBH in secondary forest and 13.2–25.7 cm in the plantation, and were allocated across the three DBH classes used for stratified upscaling (5/5/2 and 2/5/5 trees in the low, middle and high class, respectively). The sample is a structural subsample of each stand rather than a complete individual-tree inventory, so residual selectivity in the stemflow upscaling cannot be fully excluded. Ten of the twelve trees per forest type were instrumented for stemflow, and the remaining two were measured for structure only; ± SE here is the tree-level standard error (n = 12 trees), not the plot-level SE reported in Table S2.

Tree No.	Secondary forest DBH (cm)	Secondary forest height (m)	Secondary forest crown area (m2)	Plantation DBH (cm)	Plantation height (m)	Plantation crown area (m2)
1	10.3	5.0	6.00	16.5	9.5	4.40
2	15.7	5.5	3.60	22.8	10.0	3.60

Tree No.	Secondary forest DBH (cm)	Secondary forest height (m)	Secondary forest crown area (m2)	Plantation DBH (cm)	Plantation height (m)	Plantation crown area (m2)
3	5.5	2.5	0.80	13.2	8.5	2.70
4	6.2	2.5	0.25	24.7	10.0	5.00
5	3.5	3.0	0.40	15.7	9.5	3.60
6	22.0	7.5	12.00	25.7	10.5	5.00
7	4.4	2.7	0.80	16.3	8.8	3.00
8	5.8	3.7	1.50	25.6	10.0	3.96
9	11.6	4.3	2.70	16.1	9.0	3.00
10	33.5	9.6	7.50	24.8	9.0	5.00
11	15.6	7.2	3.00	13.8	8.0	2.70
12	13.2	8.8	3.06	19.6	9.8	5.00
Mean ± SE	12.28 ± 2.52	5.19 ± 0.73	3.47 ± 1.01	19.57 ± 1.40	9.38 ± 0.21	3.91 ± 0.27
SD	8.72	2.53	3.50	4.85	0.73	0.94
Range	3.5–33.5	2.5–9.6	0.25–12.0	13.2–25.7	8.0–10.5	2.7–5.0
